# Primary Hepatic Neuroendocrine Tumor Treated With Lattice and External Beam Radiation: A Case Report

**DOI:** 10.7759/cureus.89374

**Published:** 2025-08-04

**Authors:** Yeidaliz Barreto Cruz, Gordon L Grado

**Affiliations:** 1 Radiology-Oncology, Universidad Autónoma de Gualajara, Guadalajara, MEX; 2 Radiation Oncology, Southwest Oncology Centers, Scottsdale, USA

**Keywords:** external beam radiation, gastrointestinal neuroendocrine tumor, lattice, primary liver neuroendocrine tumor, radiology oncology

## Abstract

Neuroendocrine tumors (NETs) originate from neuroendocrine cells, which receive neural input and play a key role in transmitting signals by secreting bioactive substances such as monoamines (e.g., serotonin), peptides (e.g., somatostatin), and hormones (e.g., insulin) that regulate a wide range of physiological functions. While NETs in the liver are typically metastatic, often originating from other sites in the gastrointestinal (GI) tract, they can, in rare cases, manifest as primary tumors in the liver. These are referred to as primary hepatic neuroendocrine tumors (PHNT). We present the case of a 62-year-old male patient diagnosed with PHNT confirmed through imaging and immunohistochemistry analysis. Given the rarity of this diagnosis, there are no established treatment guidelines for PHNT. In this case, lattice radiotherapy (LRT) via TomoTherapy (TomoTherapy, Inc. in Madison, WI) was selected as the first-line treatment approach for the tumor.

## Introduction

Neuroendocrine tumors (NETs) arise from neuroendocrine cells, which exhibit characteristics of both nerve cells and endocrine cells, enabling NETs to receive signals from the nervous system as well as having the capacity to synthesize and secrete hormones.

These tumors are generally slow-growing and can develop in a variety of organs, including the gastrointestinal (GI) tract, pancreas, lungs, gallbladder, thymus, thyroid gland, gonads, and skin, with the GI tract accounting for 55% of existing cases [1]. 

Hepatic NETs commonly arise as metastasis from distal sites, while primary hepatic neuroendocrine tumors (PHNETs) are exceedingly rare, with only approximately 200 cases reported worldwide as of 2022. Although PHNETs do not exhibit a clear demographic pattern, they are commonly diagnosed in patients aged 47-60 years [2].

A suspected diagnosis of NETs should be followed by a comprehensive evaluation, including a detailed history, physical examination, biochemical testing, radiographic imaging, and functional imaging such as gallium-68 DOTATATE PET/CT. Metastasis should be ruled out, and patients should be evaluated for the presence of carcinoid syndrome [3]. Common symptoms include flushing (90%), diarrhea (70%), and wheezing (15%). Diagnosis is supported by a urinary 5-hydroxyindoleacetic acid (5-HIAA) test, which measures the main metabolite of serotonin, as well as chromogranin A, a tumor marker commonly elevated in NETs [4]. These diagnostic complexities make PHNET a diagnosis of exclusion.

Although no standard treatment has been established for PHNETs, management typically involves a multidisciplinary approach, with surgery being the only curative option [5]. The use of radiation therapy, either pre- or postoperatively, has been insufficiently studied in this context.

The treatment selected for this case was lattice radiotherapy (LRT) administered via Hi-Advanced Radiotherapy (Hi-ART) TomoTherapy (TomoTherapy, Inc. in Madison, WI), a first-generation device for helical intensity-modulated radiotherapy that allows image guidance using megavolt-computed tomography [6]. This case highlights the use of radiotherapy as a non-surgical approach for a patient with biopsy-confirmed PHNET who declined surgical intervention.

## Case presentation

A male patient in his early 60s presented with a diagnosis of PHNET, a rare entity typically diagnosed by exclusion. His symptoms began approximately eight to nine years prior to the diagnosis, with chronic right-sided back pain that radiated into the hip and right thigh. The pain was described as constant and not responsive to pain medication, for which the patient did not seek any medical attention at that time. An incidental hepatic lesion, approximately 1 cm in size, was discovered on CT imaging, although its relationship to the patient’s back pain was unclear. At that time, bilirubin, alkaline phosphatase, alanine aminotransferase, and hepatitis panels were all within normal range, as well as negative viral panels. A follow-up CT scan performed one year later showed that the lesion had increased in size to 1.5 x 1.0 cm, and no intervention was performed. Five years after the second CT scan, a follow-up CT scan revealed even further growth, with the lesion measuring 4.5 cm.

Histopathologic analysis performed at an outside institution confirmed the diagnosis of a well-differentiated NET. Microscopic examination of the sample revealed that the specimen was composed of a large discohesive nest of malignant cells with plasmacytoid features, characterized by dense nuclei with a moderate amount of eosinophilic cytoplasm. There was no significant nuclear polymorphism, mitotic activity, or necrosis observed. Several immunohistochemical stains were performed with adequate controls, and the neoplastic cells were found to be diffusely positive for synaptophysin, chromogranins, and CD56. Additionally, the tumor was positive for CDX2 and CK19, which are transcription factors and cytokeratins, respectively, commonly associated with GI epithelial origin, helping to further classify the tumor's site of origin [7, 8]. Additionally, Ki-67 showed a proliferation index of 3.7%. Based on these findings and in accordance with the World Health Organization (WHO) classification of 2017 [9], the neoplasm was diagnosed as a Grade 2 well-differentiated NET. 

**Figure 1:**
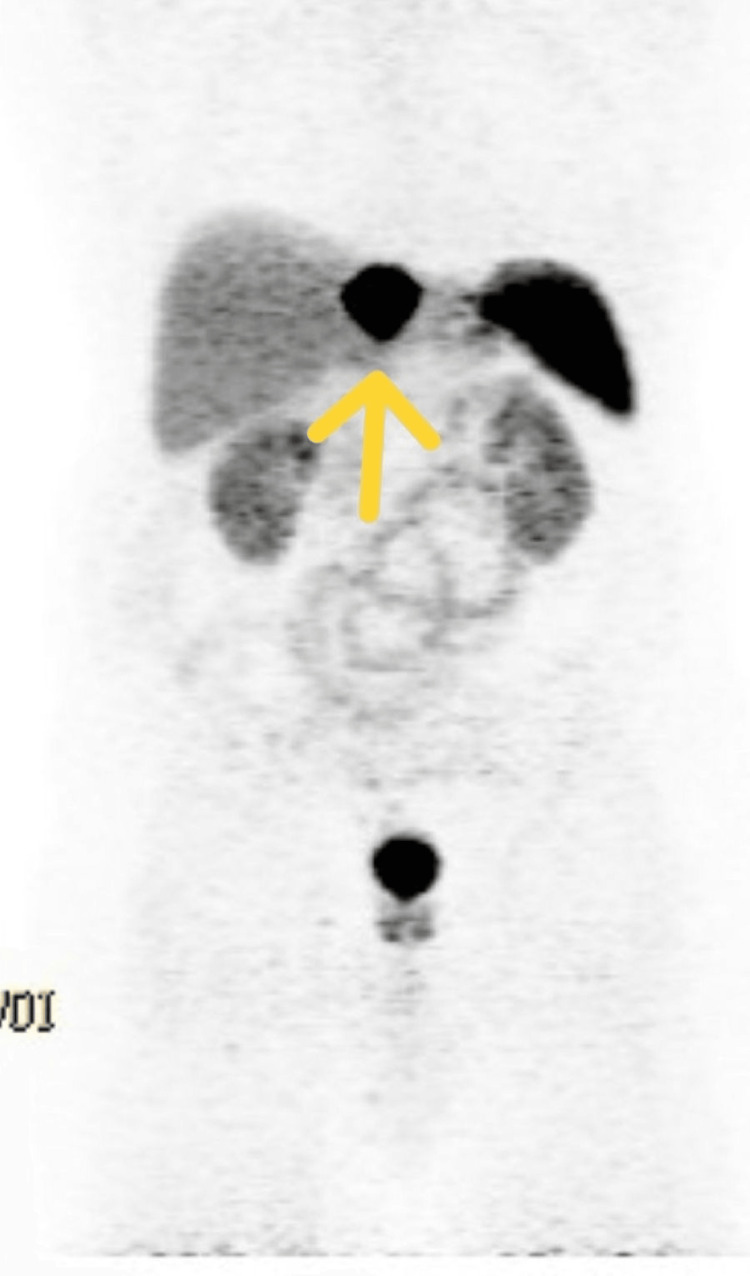
Gallium-68 DOTATATE PET/CT scan demonstrating intense uptake in primary hepatic neuroendocrine tumor (PHNET) Coronal view of a gallium-68 DOTATATE PET/CT scan showing intense radiotracer uptake (standardized uptake value (SUV) max 205) in a 4.5 × 4.0 cm hypermetabolic lesion located in the left hepatic lobe (indicated by yellow arrow). No evidence of other foci of uptake was observed, supporting the diagnosis of a solitary, well-differentiated PHNET. The scan confirmed somatostatin receptor expression and helped guide non-surgical treatment planning.

Following this diagnosis, the patient underwent a gallium-68 DOTATATE PET/CT scan, which measured the mass as 4.5 x 4 cm with a maximum standardized uptake value (SUV) of 205 (Figure 1). Subsequently, the patient underwent both an upper endoscopy and a colonoscopy, neither of which revealed any abnormalities. Serum analysis showed alpha-fetoprotein and chromogranin were within normal limits. The results for 24-hour 5-HIAA were normal.

These results and the tumor classification were thoroughly discussed with the patient, who agreed to proceed with treatment using LRT delivered via the TomoTherapy platform. LRT consisted of 27 Gy administered in three fractions to high-dose lattice vertices, followed by conventionally fractionated TomoTherapy at 1.8 Gy per fraction for 25 additional fractions.

**Figure 2:**
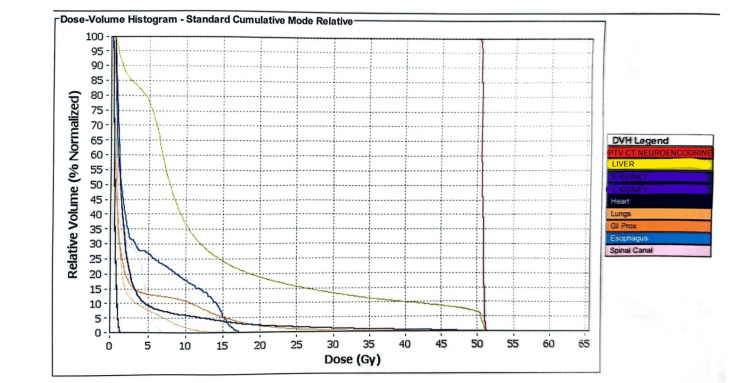
Dose-volume histogram (DVH) for planning target volume (PTV) and organs at risk (OAR) in lattice radiotherapy treatment. The PTV (red line) shows uniform coverage with a maximum dose of 51.3 Gy and an average dose of 50.7 Gy. OAR received low doses, including a liver mean dose of 13.3 Gy, spinal canal 7.1 Gy, right kidney 0.5 Gy, and heart 2.9 Gy. These values reflect effective target coverage while maintaining acceptable constraints to surrounding tissues, consistent with spatially fractionated lattice radiotherapy principles.

**Figure 3:**
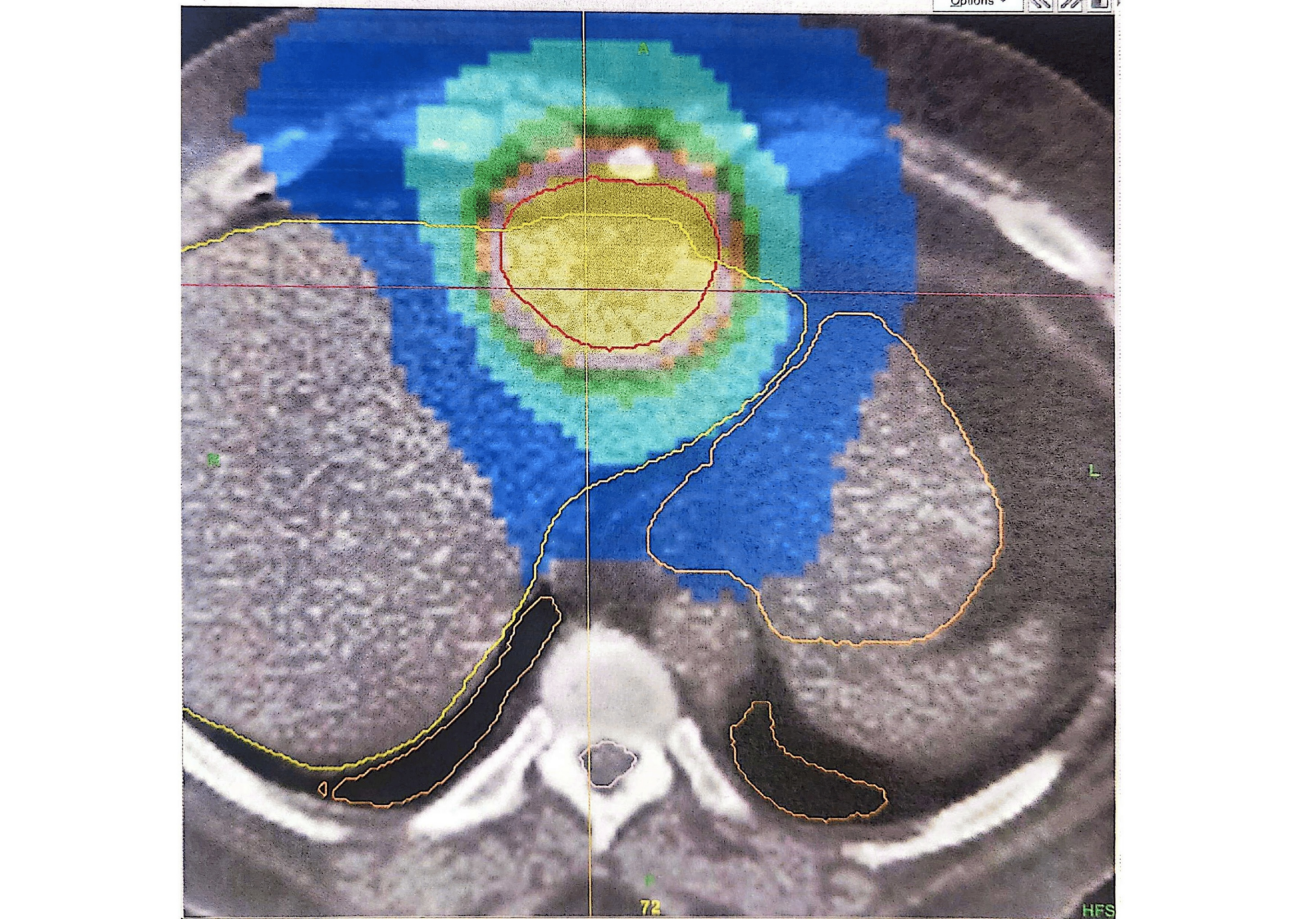
CT axial slice with isodose distribution Axial CT slice demonstrating isodose distribution from the radiation treatment plan. The central high-dose region (yellow/red) represents the lattice radiotherapy vertices within the planning target volume. Surrounding organs at risk were contoured and received lower doses, illustrating spatial fractionation and conformality achieved using the TomoTherapy platform.

Treatment planning details are illustrated in the isodose distribution (Figure 2), demonstrating dose conformity around the target volume, and in the dose-volume histogram (DVH) (Figure 3), which highlights target coverage and sparing of adjacent organs at risk (OAR).

Three months after lattice treatment, gallium-68 DOTATATE PET/CT showed a decrease in size to 4 x 4 cm with SUV of 45. The patient denied experiencing systemic symptoms during or after completing treatment.

## Discussion

Approximately 80% of NETs found in the liver are metastatic, originating from other sites, while only about 0.4% of all NETs are primary tumors arising in the liver [10]. The etiology of PHNETs is unclear, but Hu et al. outline three hypotheses to explain the origin of PHNETs: the first is that it may develop from ectopic tissues with endocrine functions; the second is that it could result from the proliferation of neuroendocrine cells in the epithelium of intrahepatic capillary bile ducts; and the third is that it may originate from pluripotent stem cells in the liver [11].

Given the rarity of PHNETs, other hepatic lesions were initially considered in the differential diagnosis. These included metastatic NETs from unknown primaries, cholangiocarcinoma, hepatocellular carcinoma, and benign hepatic lesions such as focal nodular hyperplasia or adenoma. However, extensive negative imaging and endoscopic workup, combined with immunohistochemical findings (positive CDX2, CK19, synaptophysin, chromogranin, and CD56), and the absence of extrahepatic disease on gallium-68 DOTATATE PET/CT supported the diagnosis of a solitary, well-differentiated PHNET.

Our patient presented solely with chronic back pain and lacked systemic symptoms typically associated with carcinoid syndrome. Laboratory evaluation revealed normal levels of chromogranin A and 5-HIAA. Gallium-68 DOTATATE PET/CT imaging demonstrated a large hepatic lesion with an initial SUV of 205, which declined to 45 at three-month follow-up. Although DOTATATE SUV may reflect somatostatin receptor expression, its correlation with treatment remains poorly understood. There are currently no validated PET-based metabolic response criteria for PHNET using DOTATATE imaging, and SUV interpretation can be subject to interobserver variability [12].

Reported symptoms of PHNETs are variable, ranging from incidental findings to presentations with significant mass effect. For example, Sakharuk et al. describe a case of a 57-year-old woman with abdominal pain and weight loss, whose imaging revealed a centrally necrotic liver mass causing compression of the biliary tree and adjacent organs. In contrast, our patient’s tumor was large but asymptomatic aside from mild back pain and lacked evidence of local invasion or obstruction [13].

Literature on treatment and guidelines is limited, but in a case series compiled by Wei Li et al., data were collected from seven patients diagnosed with PHNETs over 10 years. Of these, five patients underwent surgical resection, one underwent transcatheter arterial chemoembolization (TACE), and one received conservative treatment. Of the patients who underwent surgery, two experienced tumor recurrence, as did the patient who received TACE [14]. 

During discussions with the patient, radiation monotherapy was chosen after the patient declined invasive procedures. This decision was based on the absence of severe symptoms and the lack of obstruction. Radiation therapy was explored as a potential treatment for PHNETs to contribute to the limited body of literature on non-surgical management of PHNET.

In this case, LRT was selected due to the lesion’s large size and central location within the liver. LRT is a form of fractionated radiation therapy and represents a promising approach for the treatment of larger tumors. It allows for the simultaneous delivery of high ablative doses to selected sub-volumes within the tumor, known as vertices, while surrounding tissues and OAR are exposed to significantly lower doses. Treatment planning involves generating a three-dimensional lattice within the planning target volume (PTV), creating alternating regions of high-dose "peaks" and low-dose "valleys." This spatial modulation makes LRT particularly well-suited for tumors larger than 5 cm, which may not be ideal candidates for conventional stereotactic body radiation therapy (SBRT), while also aiming to reduce treatment-related toxicity [15]. As Duriseti et al. (2020) explain, lattice treatment creates high-dose islands within a sea of lower doses covering the entire tumor, making it ideal for large or deep-seated tumors near critical structures [16].

However, this study has several limitations. The short follow-up period limits the ability to evaluate long-term treatment efficacy and monitor for disease progression. Additionally, the rarity of PHNET and the lack of comparable cases treated with radiotherapy restrict the generalizability of these findings. The radiation treatment plan was not fully detailed, which may hinder reproducibility in future studies. Furthermore, post-treatment liver function and hematologic data were not available, preventing a comprehensive assessment of treatment-related toxicity. Routine collection and reporting of these parameters should be prioritized in future cases to better evaluate safety and tolerability.

## Conclusions

Although the optimal time frame for evaluating maximum treatment response and recurrence remains unclear, follow-up imaging at three months demonstrated a modest reduction in tumor size and a substantial decrease in SUV. While these findings may suggest reduced metabolic activity, their clinical significance remains uncertain. The short duration of follow-up and lack of long-term outcome data limit the ability to draw definitive conclusions about treatment efficacy. Nonetheless, this case highlights LRT as a potentially effective noninvasive option for patients who are not surgical candidates. Further studies are needed to evaluate the long-term efficacy and role of radiotherapy in the management of PHNET.

## References

[1] Ahmed M (2020). Gastrointestinal neuroendocrine tumors in 2020. World J Gastrointest Oncol.

[2] Elayan A, Batah H, Badawi M, Saadeh A, Abdel Hafez S (2022). Primary hepatic neuroendocrine tumor: a case report and literature review. Cureus.

[3] Cloyd JM, Ejaz A, Konda B, Makary MS, Pawlik TM (2020). Neuroendocrine liver metastases: a contemporary review of treatment strategies. Hepatobiliary Surg Nutr.

[4] Gade AK, Olariu E, Douthit NT (2020). Carcinoid syndrome: a review. Cureus.

[5] Yang K, Cheng YS, Yang JJ, Jiang X, Guo JX (2015). Primary hepatic neuroendocrine tumor with multiple liver metastases: a case report with review of the literature. World J Gastroenterol.

[6] Kurosaki H, Hirayama K, Takahashi M, Uematsu M, Tate E (2022). Tomotherapy: comparison of Hi-ART, Tomo-HD, and Radixact. Cureus.

[7] Ilie-Petrov AC, Cristian DA, Grama FA (2024). Evaluation of the immunohistochemical scoring system of CDX2 expression as a prognostic biomarker in colon cancer. Diagnostics (Basel).

[8] Jain R, Fischer S, Serra S, Chetty R (2010). The use of cytokeratin 19 (CK19) immunohistochemistry in lesions of the pancreas, gastrointestinal tract, and liver. Appl Immunohistochem Mol Morphol.

[9] International Agency for Research on Cancer (2017). WHO Classification of Tumours of Endocrine Organs; WHO Classification of Tumours, 4th Edition, Volume 10.

[10] Meng XF, Pan YW, Wang ZB, Duan WD (2018). Primary hepatic neuroendocrine tumor case with a preoperative course of 26 years: a case report and literature review. World J Gastroenterol.

[11] Hu HX, Yu T (2019). Primary hepatic neuroendocrine tumors: a case report. Medicine (Baltimore).

[12] Farhadi F, Saboury B, Jones E (2019). How to interpret and report ⁶⁸Ga‑DOTA‑TATE PET/CT imaging. J Nucl Med.

[13] Sakharuk I, Harner A, McKenzie J, Arfa A, Ullah A, Belakhlef S, Kruse J (2022). Large primary neuroendocrine tumor of the liver in a 57-year-old female presenting with MSSA bacteremia. Am Surg.

[14] Li W, Zhuang BW, Wang Z (2016). Case report of contrast-enhanced ultrasound features of primary hepatic neuroendocrine tumor: a CARE-compliant article. Medicine (Baltimore).

[15] Iori F, Cappelli A, D'Angelo E (2023). Lattice radiation therapy in clinical practice: a systematic review. Clin Transl Radiat Oncol.

[16] Duriseti S, Kavanaugh J, Goddu S (2021). Spatially fractionated stereotactic body radiation therapy (lattice) for large tumors. Adv Radiat Oncol.

